# The Relationship between Coaches’ and Athletes’ Competitive Anxiety,and their Performance

**Published:** 2013

**Authors:** Mahmoodreza Mottaghi, Alireza Atarodi, Zahra Rohani

**Affiliations:** 1 Department of Basic Sciences, School of Medicine, Gonabad University of Medical Sciences, Gonabad, Iran.; 2 Faculty Member, Department of Basic Sciences, School of Medicine AND Social and Health Development Research Center, Gonabad University of Medical Sciences, Gonabad, Iran.

**Keywords:** Competitive Anxiety, Coach, Futsal, Performance, Player

## Abstract

**Objective:** This study was performed to survey the relationship between coaches’ and athletes’ competitive anxiety, and their performance.

**Methods:** This is a descriptive correlational study performed using a demographic questionnaire, an athletic performance checklist, and sport competition anxiety test designed by Martens consisting of 15 questions. The study population consisted of 540 players and 60 coaches from 60 futsal teams (5 main players, 4 reserves, and 1 coach for each team). All of the players and the coaches were surveyed in a census method and no sampling was done. The data were analyzed by SPSS software, using chi-square, and Pearson correlation coefficient test.

**Results: **The results showed a positive significant relationship between the coaches’ anxiety level and sport competition anxiety level in the athletes (p = 0.019, r = 0.56). It also showed that there was a negative significant relationship between the coaches’ anxiety level and performance level of the athletes (p = 0.012, r = -0.80). A negative significant relationship was also demonstrated between the athletes’ competitive anxiety level, and their athletic experiences (p < 0.001, r = -0.45) and age (p = 0.001, r = -0.37).

**Conclusions:** Coaches and officials should consider sport competition anxiety among athletes before and during competitions. Formal and planned competitions, training sessions, and preparation practices can be a major factor assisting to decrease athletes' anxiety.

**Declaration of interest:** None.

## Introduction

Anxiety is one of the most important and interesting areas of focus in sport psychology that has attracted many researchers who have mostly considered athletes and coaches anxiety experiences (1). 

Sport psychology is one of the sub-branches of psychology and is affiliated to both psychology and sport sciences. One of the main topics considered in sport psychology that can affect the performance of athletes especially in championships is anxiety level before competition and its effect on athletes' moods and locomotors skills. Generally athletes are anxious for many and various reasons, such as the importance of sporting success, or the difference between their abilities and capabilities needed for their sport, and these factors can have a negative effect on their performance. Research findings show that excitement and arousal, or anxiety can affect athletic performance (2). 

“Considering the importance of the coach in determining the quality and success of an athlete's sport experience, surprisingly little research exists that identifies optimal coaching behaviors and factors that influence the effectiveness of particular behaviors. It is proposed that coaching effectiveness is mediated by athletes' perception and recall. Overt coaching behaviors are perceived and given meaning by each athlete resulting in an attitude toward both the coach and the sport experience. Similarly, it has been suggested that an individual's perception of another's behavior is more important than the behavior itself in determining one's feelings or actions toward other person” (3).

Therefore, it is well recognized that the level of performance and function of athletes in competitive sports is influenced by some psychological factors such as personality traits, competitive anxiety, and coping strategies. The relationship between anxiety and level of performance in various sports has been the subject of many studies. If athletes do not have sufficient coping skills to deal appropriately with these situations, they will be exposed to risks of poor performance, failure, and even serious injury (4).

Competitive anxiety can affect athletes’ feelings, thus it can impair athletes’ performance and function. Preliminary studies of anxiety were based on the presumption that anxiety was one-dimensional. For example, Zamani and Moradi realized that competitive anxiety for young participants was higher in individual sports than team sports, and also higher in individual combat sports in comparison to individual non-combat sports (5).

Competitive anxiety is a kind of anxiety formed in competitive situations in sports. One of the major causes of competitive anxiety are social factors. Continual pressure on a young athlete resulting from expectations of parents, coaches, and teammates can generate much anxiety; coaches can have the main and most influential role in this respect in youth sports. Sedarati has shown, in his study, that competitive anxiety is caused due to unrealistic beliefs, employing someone who thinks he will have a poor performance, worrying that someone will guess they are anxious, performance experience below expected levels in his previous match, a person with mental challenges, catastrophic and previous problems, and individual beliefs related to the implementation of sport skills. Athletes who have high sport performance anxiety seem to be especially more sensitive to fears of failure resulting in negative social- and self-evaluation. Generally, the relationship between anxiety and athletic performance is expressed by stimulation theory and the hypothesis of reverse or (u) theory (2).

Zamani and Moradi identified years of experience as the best predictor of cognitive component; one or more factors related to negative expectations of athletes can affect their performance and lead to a negative self-assessment. Moreover, they identified trait anxiety as the best predictor of physical component; one factor related to anxiety is physiologic elements emerging from direct nerve stimulation. Physiological responses present as palpitation, hyperpnea, hand-sweating, muscles stiffness, and etcetera among wrestlers. Zamani and Moradi also found that there was no significant difference between gender and the different levels of anxiety before and during a volleyball match in volleyball players (5). 

The findings of the current study emphasized the relationship between anxiety and performance, and the negative impact of anxiety on athletic performance. Abolghasemi et al. demonstrated a significant negative correlation between competitive anxiety and sport performance. They also showed that cognitive anxiety and physical anxiety of athletes increased as approaching the race, but their confidence decreased. A significant negative correlation was also found between competitive anxiety and athletic performance (6).

Arianpooran and Abolghasemi studied the relationship between coping strategies, goal orientation, and performance anxiety of the students with athletic performance. They found that the strongest predictor of individual athletic performance was goal orientation, and in team sports it was goal orientation and competitive anxiety (7). Sedarati concluded that the highest mean of competitive anxiety was found in chess competition athletes and the lowest was seen among basketball players (2).

Abolghasemi et al. by evaluating the effect of competitive anxiety on athletic performance concluded that increase in competitive anxiety will result in reduction of academic achievement and athletic performance (6). Zamani and Moradi compared the state and trait of anxiety and self-confidence of male athletes and reached the conclusion that individual sport athletes had higher anxiety and less self-confidence in comparison with team sport athletes. However, no significant difference was shown between the cognitive components of the state of anxiety of both groups (5). The first studies on anxiety were based on the pre-supposition that anxiety was one-dimensional. However, some studies concluded that competitive anxiety is higher in young participants in individual sports than team sports (7). Some other studies have not supported this conclusion (8).

As another study declared, cognitive anxiety results in defeat. This theory of anxiety suggests that cognitive anxiety has a negative relationship with fulfillment, level of experience, and changes in anxiety level at different times influencing the athletes’ performance (9).

Each athletic club or organization can achieve its expected or predetermined goals when it has expert and skilled instructors and coaches. Evidently, a coach can have an effective role in the athlete’s performance. In sport teams, an athlete does his/her best to perform positively and efficiently from his coach's point of view and tries to perform all of the coach’s recommendations. Considering this close relationship between a coach and an athlete, the coach should consider his athletic feelings and behavior to be enough. However, some different factors, such as club or team supervisors’ expectations and wishes, and futsal lovers and their fans' desire for them to win the game can make the coach anxious. In order to reach the goal, a coach should make appropriate and correct decisions with diplomacy and tactfulness based on his experience. It is clear that an anxious coach will not be able to make correct decisions or to plan, manage, and lead the team favorably and this can make the players anxious and depressed resulting in an unfavorable outcome. Coaches should choose the best and most appropriate way to achieve goals through understanding of physical and mental capabilities of the athletes and their subordinates. Since the sport of futsal has been accepted in the world and in the Islamic society of Iran as an attractive and popular sport, it has developed widely among children, adolescents, youth, and even adults. Therefore, we decided, as a necessity, to study psychological factors affecting athletes' traits in this field. We also aimed to answer the following questions: 

1. Is there any relationship between athletes' and coaches' anxiety level? 

2. Is there any relationship between coaches' anxiety and athletes' performance? 3. Is there any relationship between certain demographic characteristics (age, athletic experience, and etcetera) and athletes' anxiety level? 

The study was designed to reduce athletes’ anxiety by helping coaches to create a situation associated with lower levels of anxiety in athletes to show their best potential.

## Materials and Methods

This is a descriptive, correlational study performed using Martens's Sport Competition Anxiety Test and a demographic questionnaire (variables such as age and experience are factors that affect anxiety level) and a checklist for athletic performance. 

*Area of Study*

Gonabad city with an area of 10,000 km^2^ in the south of Khorasan-e-Razavi Province, 260 km from Mashhad, located at 34.35° north latitude, 58.68° east longitude, and about 1098 meters altitude above sea level with a hot and dry climate, and neighboring Southern Khorasan and Sistan and Baluchestan Provinces in Iran (10). 

*Participants*

The participants were 60 male coaches and 540 athletes (futsal players) who participated in futsal programs in Gonabad, Iran. The mean age of the coaches was 30.00 ± 2.32 years, and their mean year of futsal coaching experience was 9.00 ± 2.76 years. The mean age of the athletes was 22.00 ± 1.68 years and their mean year of futsal playing experience was 5.00 ± 1.06 years. The mean stature of the coaches was 175.0 ± 3.4 cm and of the players was 165.0 ± 2.6 cm. The mean of the coaches’ body mass index was 85.0 ± 4.7 and it was 63.0 ± 3.8 for the players. Because these games were played once a year and due to the great desire of youth, fans, coaches, and also players themselves to watch these games, we thought it was necessary to conduct this kind of game in order to experience the coaches' and athletes’ anxiety closely. Since, another similar research had previously been done on individual sport by other investigators we tried to avoid repetition and thus performed this study.

*Procedure*

The study population consisted of all coaches and futsal players that participated in Ramazan Futsal Cup in Gonabad in 2010. According to the accepted rules, each team included 10 members (9 players and 1 coach); thus, a total of 540 futsal players and 60 coaches in 60 teams formed the study population. There were 10 members per team (5 main players, 4 reserve players, and 1 coach) that participated in the study. No sampling was performed and all of the futsal players and coaches were surveyed in a census method. The questionnaires were submitted to the players before the game started. For a higher validity of the results, each team was tested 3 times during three different games, and the mean score was derived. The subjects participated voluntarily and were ensured about the privacy of the data. We also obtained the coach's permission. 

*Measures*

Sport Performance Anxiety: The main measurement instrument in this study was sport competition anxiety test (SCAT) provided by Martens (1990) for measuring competitive anxiety level. It consisted of 15 questions; 10 items that were the main questions and were scored normally, and 5 others that were neutral questions and were not scored. The items were answered in a 3-point Likert scale of 1. Seldom, 2. Sometimes, 3. Often. The total score was estimated 10 for low anxiety and 30 for high anxiety. The questions 8, 9, 12, 14, and 15 were related to evaluation of physical competitive anxiety level, and questions 2, 3, 5, 6, and 11 were for cognitive competitive anxiety evaluation. The questions 1, 4, 7, 10, and 13 were not scored, and were placed in the context only for questionnaire control. The questions 6 and 11 were scored in reverse and the rest of the questions were scored normally from 1 to 3. Total score was between 10 and 30. The higher the obtained score, the more risk of being anxious before the match. Sports performance checklist also included 18 items, which were completed by the coach, regarding the athletic performance and outcomes of athletes and sports teams in Gonabad province (in the present study internal consistency of the checklist was considered 0.79). This questionnaire had been used previously by Hakkak, and its validity and reliability had been approved. Cronbach's alpha coefficient of this questionnaire was 0.89 (11). Moreover, its time reliability had been found suitable (0.98) by Martens through test-retest. 

*Statistical analysis*

The data were analyzed by SPSS for Windows (version 19; SPSS Inc., Chicago, IL., USA), and distribution of data was expressed in mean and standard deviation. In order to test the research hypotheses Pearson correlation coefficient test was used. 

*Ethical considerations*

The present study was approved by the Ethics Committee of Gonabad University of Medical Sciences. The participants received oral information about the study. Their data were kept anonymous. All of them agreed to voluntarily participate in the study. All the participants were informed of the study objectives and how to complete the questionnaires. Before the interview, a written informed consent was obtained from all of the participants, they were assured that the information would remain confidential, and they were told that they would be allowed to leave the study at any stage if they did not like to continue.

## Results

The mean age of participating players was 22.00 ± 1.68 years and the mean age of the coaches was 30.00 ± 2.32 years. The mean year of the athletes’ experiences was 5.00 ± 1.06 years and it was 9.00 ± 2.76 years for the coaches. The mean anxiety score of the players was 16.79 ± 2.10 and it was 17.20 ± 2.21 for the coaches. The results are shown in tables 1 and 2.

Table 1 suggests that there is a positive significant relationship between the coaches' and the athletes' anxiety (p = 0.019, r = 0.56). There was also a negative significant relationship between the coaches' anxiety and the athletes' performance (p = 0.012).

**Table 1.** The relationship between the coaches' and the athletes' anxiety and their performance

	**Coaches' anxiety**	
	**Correlation coefficient**	**P-value**
**Athletes' anxiety**	0.56	0.019
**Athletes' performance**	-0.80	0.012

**Table 2:** The relationship between the athletes' competitive anxiety and their performance, age, and experience

**Variables**	**Athletes' anxiety**	
	**Correlation coefficient**	**P-value**
**Athletes' performance**	-0.81	0.010
**Athletes' experience**	-0.45	< 0.001
**Athletes' age**	-0.37	< 0.001

Table 2 shows that there was a negative significant relationship between the athletes' competitive anxiety and their performance (p = 0.010). It also demonstrates that there was a negative significant relationship between the coaches' anxiety levels and the athletes' performance level (p = 0.010,r = -0.8). Furthermore, a negative significant relationship was observed between the athletes' competitive anxiety level and their experiences (p < 0.001, r = -0.45), and their age (p < 0.001, r = -0.37).

## Discussion

The purpose of this study was to evaluate the relation between coaches' anxiety, and athletes' anxiety and their performance. The results showed that there was a significant relationship between the coaches' anxiety level and the athletes' anxiety level. In other words, coaches’ anxiety has a meaningful impact on athletes’ anxiety changes. This is consistent with the studies by Payne (12), Hanton et al. (13), Lorimer and Westbury (14), Coudevylle et al. (15), Dominikus et al. (16), and Esfahani and Gheze Soflu (17) who believed that personal and psychological characteristics and environmental conditions play a major role in development of anxiety. 

Athletes’ expectations of themselves, their goals, their images of their coaches, and their teammates’ expectations of them are factors effective in causing anxiety. For example, in a young athlete, constant pressure from his coach for a successful competition can cause high levels of anxiety. On the other hand, coachs’ support in a way that gives athletes the opportunity to understand acceptable defeat might reduce anxiety and lead to improvement of athletic performance. 

If a match is very important for coaches and athletes, individuals feel that they are less capable than their opponent, and coaches or athletes have unrealistic fear of confrontation with the opponents the anxiety level of athletes will increase and it will have a negative impact on their memory ultimately decreasing their performance. In other words, fear of failure, sense of inability, fear of losing control of the game, and psychological stress will increase athletes' anxiety level (18). There was a negative significant correlation between the coaches anxiety and the athletes' performance. 

The second hypothesis, which suggests a significant relationship between coaches' anxiety and athletes' performance, was approved. Negative correlation meant that as the Coachs’ anxiety scores increased, the athletes' performance (final result) became weaker. These results are consistent with the results of the studies by Bray and Martin (19), Mellalieu et al. (20), Avramidou et al. (21), and Ak et al. (22). They concluded that concern, which is the source of anxiety, not only involved the mind but also affected the whole body by the reactions resulting from it that lead the person to express special behavior. If the excitement of racing is accompanied with fear from anxious coach's voice, which highlights athletes' misperformance, it can also increase anxiety of the team based on the importance of the match. 

We can probably say that the coach's sincerity and principled relationship with the athletes can create a friendly and safe environment for athletes. This can in turn prevent mental concerns and also help athletes to focus more on sports activities. The coaches' anxious behavior, and undue and excessive feedback can cause mental imbalance or disturb the athletes’ focus leading to poor performance by the individual and team (18). 

The results also showed that there was an inverse and significant relationship between the athletes’ anxiety and their performance. This was consistent with the results of the studies by Mamassis and Doganis (23), Loupos et al. (24), Pigozzi et al. (25), and Bawa (26). They had concluded that activity, working memory, and individual's attention are impaired in high anxiety conditions. Anxiety is occasionally severely disabling, resulting in chronic psychiatric invalidism, paramnesia, negligence, or inattentiveness and distraction among the youth. Memory means to remember the events which have previously happened and to access them, or to pay attention and make an image or phantasm, to save it in the mind and make the memory accessible. 

Anxious people have memory but cannot use it well, and are inattentive and cannot decide perfectly resulting in a weak performance. Fear of failure or misperformance, and parents', friends', coaches', and others' assessment can adversely affect the athletes’ performance (2). 

Weinberg and Gould (27) suggested there was low anxiety in Golf, but Wilson et al. (28) suggested there was high anxiety in runners that somehow showed a contradiction. This contradiction is that performance of sports with low complexity, such as running, is facilitated with higher levels of anxiety, but implementation of complex skills that require much concentration and precision can be performed better with lower levels of anxiety. If their performance anxiety is higher than optimal level it will disrupt their performance. Ultimately, in more complex skills, the average level of anxiety for optimum performance of the skills should be lower, and high anxiety reduces performance. Contrariwise, in skills with lack of complexity, such as weightlifting, the favorable anxiety levels are higher (2). 

However, some researches show opposite findings. The results showed that there was a significant reverse relationship between the sports experience and the athletes' anxiety. This has been previously stated by Bawa (26), Weinberg and Gould (27), Iizuka et al. (29), and Grossbard et al. (30) in their researches. In the literature review, except for the recent study, an opposite result was not observed. Considering conducted researches, it is likely that experienced athletes, often experience various levels in sports competitions, avoid psychological problems caused by negative and unrealistic thoughts and ideas, and have less anxiety in comparison to non-athletes or less experienced athletes. In other words, regular exercise and official tournaments help the athlete pay less attention to disturbing stimuli and think more in a way that can be effective in their success. Therefore, practice and experience makes athletes identify the sources of and methods that reduce anxiety. 

Hence, it is known that someone who is more skilled is also more successful. However, other research hypotheses showed a statistical relationship between age and anxiety level of the athletes. The results of this study showed that there was a significant inverse (negative) relationship between the athletes' anxiety levels and their age. In a sense, anxiety is reduced with increase in athletes’ age. In the studies by Cartoni et al. (31) and Pears (32), it was concluded that when an athlete’s age increases, their anxiety is reduced; which was consistent with our findings. 

Since athletes age differentiation in these researches is a maximum of three years, and the subjects almost possess the same conditions in terms of physical and mental growth, the results of these studies’ cannot be definite and valuable. It would be better if broader age ranges were considered to check this variable. Thus, we can conclude that the higher the athletes' age is the lower their anxiety will be. 

A survey by Neil et al. was done on 115 male rugby union performers (elite: n = 65, non-elite: n = 50) aged between 18 and 36 years. They concluded that elite and non-elite athletes differed in their use of psychological skills to cope with their experiences of symptoms associated with competitive anxiety (33). 

A survey was performed by Esfahani and Gheze Soflu in Iran on male and female volleyball players (n = 214). They used a questionnaire, distributed among the study population just 30 minutes before a competition, aiming to compare pre-competition anxiety, and anger between female and male volleyball players who were college students. They concluded that there was a significant difference in all pre-competition anxiety subscales: cognitive state anxiety, somatic state anxiety, and self-confidence. However, it did not show a significant difference in trait of anger. Nevertheless, it showed a significant difference in state of anger and the expression of anger (17). 

A research was performed by Singh et al. in India comparing pre-competitive and post-competitive anxiety of basketball players of Punjab University. The study population was 30 players of 18-25 years of age (15 of each sex). They found that there was a significant difference in anxiety levels of pre-competitive anxiety and post-competitive anxiety among the male and female basketball players. Finally, they recommended that coaches design appropriate training programs to help athletes reduce their anxiety levels, and enhance their performance and function through acquiring effective and efficient sport skills (34). 

These studies (17, 33, 34) are in accordance with the present study; they showed there was a significant relationship between the coaches' and the athletes' anxiety, and between the coaches' anxiety and the athletes' performance. Therefore, any type of anxiety can affect athletes’ performances and activities. 

In another study aiming to evaluate the relationship between goal orientation and competitive anxiety in female athlete students engaging in individual and team sports Morgan’s table was used. One hundred and twenty athletes were randomly selected from team sports and 80 athletes were selected from individual sports. The results showed no significant relationship between task orientation and competitive anxiety in the individual sports group. Moreover, goal orientation of team sport and the individual sport athletes had no significant relationship with their competitive anxiety (35). 

The present study showed that there was a significant relationship between anxiety and the athletics performance. Since the correlation was very strong, reverse, and meaningful, it could be concluded that as the athletes’ anxiety scores increased, their performance became weaker, and their team also came down to a lower category or score in the table. Coaching anxiety affected the athletes’ anxiety and performance; high coaching anxiety decreased the athletes’ performance. In addition, the competitive behaviors of one coach can be influential on reducing athlete’s anxiety. Competitive anxiety had a stronger impact on motor skills during a competition than other psychological factors. 

Another point which emerged from the results was that in order to continue successful efforts of athletes, coaches must be aware of needs, motivations, and physical and mental characteristics of athletes and themselves. Therefore, coaches and officials should consider competitive anxiety among athletes before and during competitions. Since competitive anxiety level is changeable and very complicated in different individuals and situations, no instructions with general validity and credibility can be created (17). 

The best way for coaches and sport psychologists is to precisely select their athletes based on their anxiety and try to reduce competitive anxiety based on individual characteristics and motor skills performance (18). It seems necessary for coaches and sports psychologists to decrease anxiety through several approaches. Undoubtedly, increasing the number of formal and planned competitions, training sessions, and preparatory practices can be a major factor in reducing athletes' anxiety (26). 

A coach must be relaxed and not nervous in order to understand the match and practice conditions and control them to win the competition with appropriate planning and skill, and he should avoid anxious speeches and behaviors toward his athletes in order to implement his plans in a relaxed atmosphere. This means that along with increase in players’ anxiety, their success and performance declined. The table results showed that there was a significant relationship between the athletes’ anxiety, experience, and age. In other words, with increase in the athletes' age and experience, their anxiety became lower. 

Coaches must also provide specific exercises through correct understanding of moralities and psychological moods of all athletes, and set up counseling sessions for all members of the younger teams to make them ready during several months before the competitions. It is recommended to seriously evaluate sport competition anxiety, and try to decrease it in team players and especially coaches in order to gain positive and efficient results. It is also suggested that a similar research be conducted on coaches or women athletes and to compare their results with the present study. 
